# Therapeutic Performance of Needle Injection Versus Needle‐Free Jet Injector System for Polynucleotide Filler in Skin Rejuvenation

**DOI:** 10.1111/jocd.16595

**Published:** 2024-10-07

**Authors:** Ji Yeon Hong, Yoon Hwan Lee, Hyun‐Jo Kim, Kui Young Park

**Affiliations:** ^1^ Department of Dermatology Chung‐Ang University Hospital, Chung‐Ang University College of Medicine Seoul Korea; ^2^ CNP Skin Clinic Asan Korea

**Keywords:** needle‐free jet system, polynucleotide, pore, rejuvenation, wrinkle

## Abstract

**Background:**

Polynucleotide (PN) filler often causes pain and can lead to delivery inaccuracies when applied via intradermal injection using a traditional needle.

**Aims:**

To evaluate the efficacy of treatment and the pain during the procedure using conventional needle injection versus a needle‐free jet system for intradermal PN filler application.

**Methods:**

In this split‐face clinical trial, 10 Korean subjects were enrolled. Each subject received an intradermal injection of PN filler on one side of the face and a needle‐free jet injection using CureJet on the other side. Assessments included global and 3D skin imaging at each visit. Pain intensity was evaluated using visual analogue scale (VAS) scores during the injection. Additionally, patient satisfaction and adverse events were documented.

**Results:**

Findings revealed that Global Aesthetic Improvement Scale scores and patient satisfaction were significantly higher with the CureJet compared to the needle injection method. VAS scores were notably lower on the CureJet side. Improvements in both pore and wrinkle indices were observed from baseline, with a more pronounced improvement rate on the CureJet side compared to the needle injection side.

**Conclusions:**

Needle‐free injection of PN for aging skin was found to be effective in enhancing pore and wrinkle improvement, while reducing associated discomfort.

## 1 Introduction

Aged skin presents various aging indicators, including loss of elasticity, epidermal thinning, diminished collagen levels, increased wrinkles, and enlarged pores [1]. Skin aging typically involves reduced collagen production, structural alterations in the collagen fiber network, and accumulation of degenerated elastic fibers in the papillary and mid‐dermis [2]. Due to the significant social implications of skin aging, there is an increasing demand for minimally invasive dermal rejuvenation procedures with short downtime. Numerous facial fillers and skin boosters have been developed to meet this need [3].

Purified polynucleotide (PN) derived from the germ cells of salmon and other fish is widely utilized for this purpose [4]. Research indicates that PN aids in the proliferation of dermal fibroblasts and stimulates angiogenesis, contributing to effective wound healing [5, 6]. Additionally, previous studies have shown that intradermal PN filler injections enhance skin elasticity and collagen synthesis by activating fibroblasts [7]. However, a significant limitation of intradermal PN injections is the substantial pain experienced by patients, even with local anesthesia. This discomfort can lead to involuntary facial movements or pressure application during the procedure, potentially increasing bleeding and impeding precise filler delivery.

In this investigator‐blind, randomized, split face study, we performed local PN injections using a conventional injection needle on one side of the aged face and a needle‐free jet system (CureJet; Bazbiomedic CO., LTD, Seoul, Korea) on the other side. Our objective was to compare the effectiveness of these two methods and the level of pain experienced during the procedure.

## 2 Materials and Methods

### 2.1 Ethics Statement

The Institutional Review Board granted approval for this study (IRB approval number: 2303‐001‐545), ensuring adherence to the Declaration of Helsinki guidelines. Informed consent was obtained from all participants prior to their involvement in the study.

### 2.2 Subjects

We conducted this study as an investigator‐blind, randomized, split‐face study. Our cohort comprised 10 healthy Korean volunteers, all over 19 years old, seeking cosmetic enhancement of their facial skin. Exclusion criteria included individuals who had received any facial skin cosmetic treatments in the preceding 2 months. These treatments included topical agents, oral medications, laser procedures, and peels, as well as those who had undergone PN treatments on their facial skin in the past 6 months.

### 2.3 Devices

This study utilized the CureJet, a needle‐free, low‐pressure jet injector with adjustable volume and pressure settings. The device operates by generating an electro‐magnetic field via a coil, which propels fluid into the skin. It offers three modes of operation: single, auto, and burst. The burst mode is subdivided into 2‐burst, 3‐burst, and 4‐burst options, based on activation frequency. Power levels range from 1 to 5 units, incrementally increasing with voltage. The operational speed, measured in hertz (Hz), varies across five settings: 1, 3, 5, 15, and 20 Hz. The volume setting, indicating fluid dispensed, increments by 0.1, with a range from 1.0 to 2.0. Each cycle delivers between 0.5 and 0.8 μL. The jet pressure, emanating from the nozzle, scales with voltage and varies between 2.21 MPa and 6.57 MPa.

Rejuran (Pharmaresearch Products, Inc., Seoul, Korea) is a transparent liquid consisting of 20 mg/mL of PN.

### 2.4 Intervention

To achieve local anesthesia prior to injection, EMLA 5% cream (lidocaine 25 mg/g, prilocaine 25 mg/g) was applied to both sides of the face for 30 min. The skin was then cleansed with chlorhexidine. Each participant were randomly assigned to receive intradermal needle injections of PN on one side of the face and microjet injections on the opposite side.

The intradermal injections utilized a 33‐gauge nano‐needle, employing a serial puncture technique. The injections were spaced about 1 cm apart. Each side of the face received 2.0 mL of filler, with 1.0 mL administered per side. For the clinical application of CureJet with PN, initial settings were Power level 5, Speed 10, and Volume 1.5 to create the jet. Subsequent settings during the procedure were Power level 2, Speed 5, and Volume 5. A 2 mL Rejuran syringe was attached as‐is to the CureJet for use.

### 2.5 Assessment

During each session, a comprehensive evaluation of the facial skin was performed by the dermatologist by face‐to‐face with all participants. The degree of improvement in facial skin aging was assessed using the Global Aesthetic Improvement Scale (GAIS). This involved two independent dermatologists who were not involved in performing the treatment assigning a GAIS score, utilizing a 5‐point grading system to categorize the level of improvement (1 = worsened, 2 = unchanged, 3 = improved, 4 = much improved, 5 = very much improved). The GAIS score was presented as the average of the two evaluators' scores.

Additionally, the efficacy of the treatment was objectively measured using a noninvasive imaging technique. Various skin rejuvenation parameters, including dilated pores, brightness, sebum levels, wrinkles, melanin content, erythema, and overall skin tone, were quantified using the Mark–Vu system.

Pain intensity was also evaluated by the participants using a visual analogue scale (VAS), which ranged from 0 (no pain) to 10 (extreme pain) during the treatments. Participants were requested to report any discomfort or adverse effects, such as erythema, edema, or bruising, both during and after the procedure. Regular physical examinations were conducted as part of safety monitoring.

Participant satisfaction was assessed immediately following the treatment and again after 2 weeks. Satisfaction levels were classified as: 1 for unsatisfied, 2 for slightly unsatisfied, 3 for neutral, 4 for slightly satisfied, and 5 for satisfied.

### 2.6 Statistical Analysis

Data analysis was conducted using SPSS version 26.0 (IBM Corp., Armonk, NY, USA). The results are presented as mean ± standard deviation. Descriptive statistics were applied to demographic data (age and sex). Due to the small sample size and the lack of normal distribution, the Wilcoxon signed‐rank test was utilized to compare differences in GAIS scores, patient satisfaction, and VAS pain scores and was employed for the analysis of skin parameters such as dilated pores, brightness, sebum, wrinkles, melanin, and erythema, as measured by the Mark–Vu system. *p* < 0.05 was considered statistically significant.

## 3 Results

### 3.1 Subjects and Efficacy Outcomes

**FIGURE 1:**
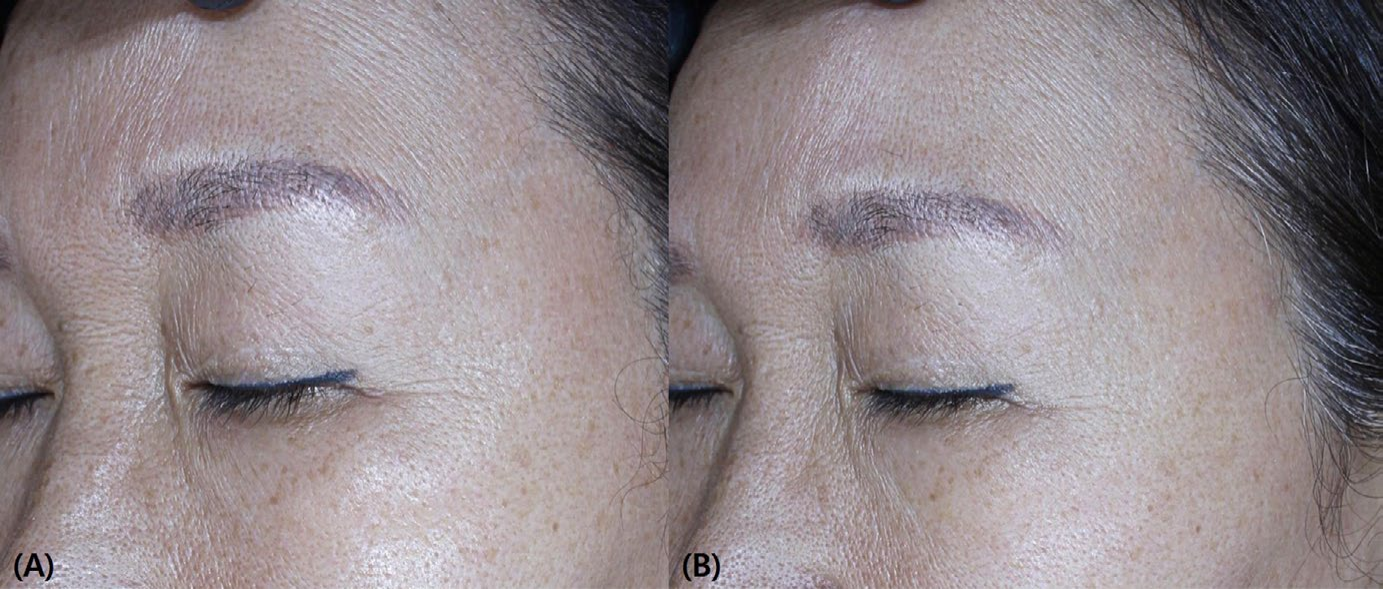
Representative case demonstrating noticeable enhancement in overall skin tone, wrinkle reduction, and smaller pore size following a 2‐weeks treatment period. (A) Before and (B) after 2 weeks of Curejet® injection.

This study enrolled 10 patients with an average age of 49.9 ± 9.47 years. The GAIS score, indicating improvement, was significantly higher in the CureJet‐treated areas (3.85 ± 0.63) compared to the manually injected areas (3.6 ± 0.52) (*p* = 0.037) (Table 1). The VAS score, reflecting pain intensity, was notably lower in the CureJet‐treated areas (2.9 ± 1.52) compared to the manually injected areas (5.4 ± 1.42) (*p* = 0.017). The overall patient satisfaction scores were also greater in the CureJet‐treated areas (4.5 ± 0.53) compared to the manually injected areas (3.9 ± 0.73) (*p* = 0.019). Figure 1 presents a representative case.

**TABLE 1:** GAIS score evaluated by two dermatologists after 2 weeks.

	Needle‐free jet injection		Conventional injection	
	Dermatologist 1	Dermatologist 2	Dermatologist 1	Dermatologist 2
Subject 1	5	4	4	4
Subject 2	5	5	5	4
Subject 3	3	4	3	4
Subject 4	4	4	4	4
Subject 5	4	3	4	3
Subject 6	4	3	3	3
Subject 7	3	4	3	3
Subject 8	3	3	3	3
Subject 9	4	3	4	3
Subject 10	5	4	5	3
Average	3.85		3.6	
SD	0.6258		0.5164	
*p* value	0.037			

### 3.2 Objective Measurement of Skin Biophysical Properties

**FIGURE 2:**
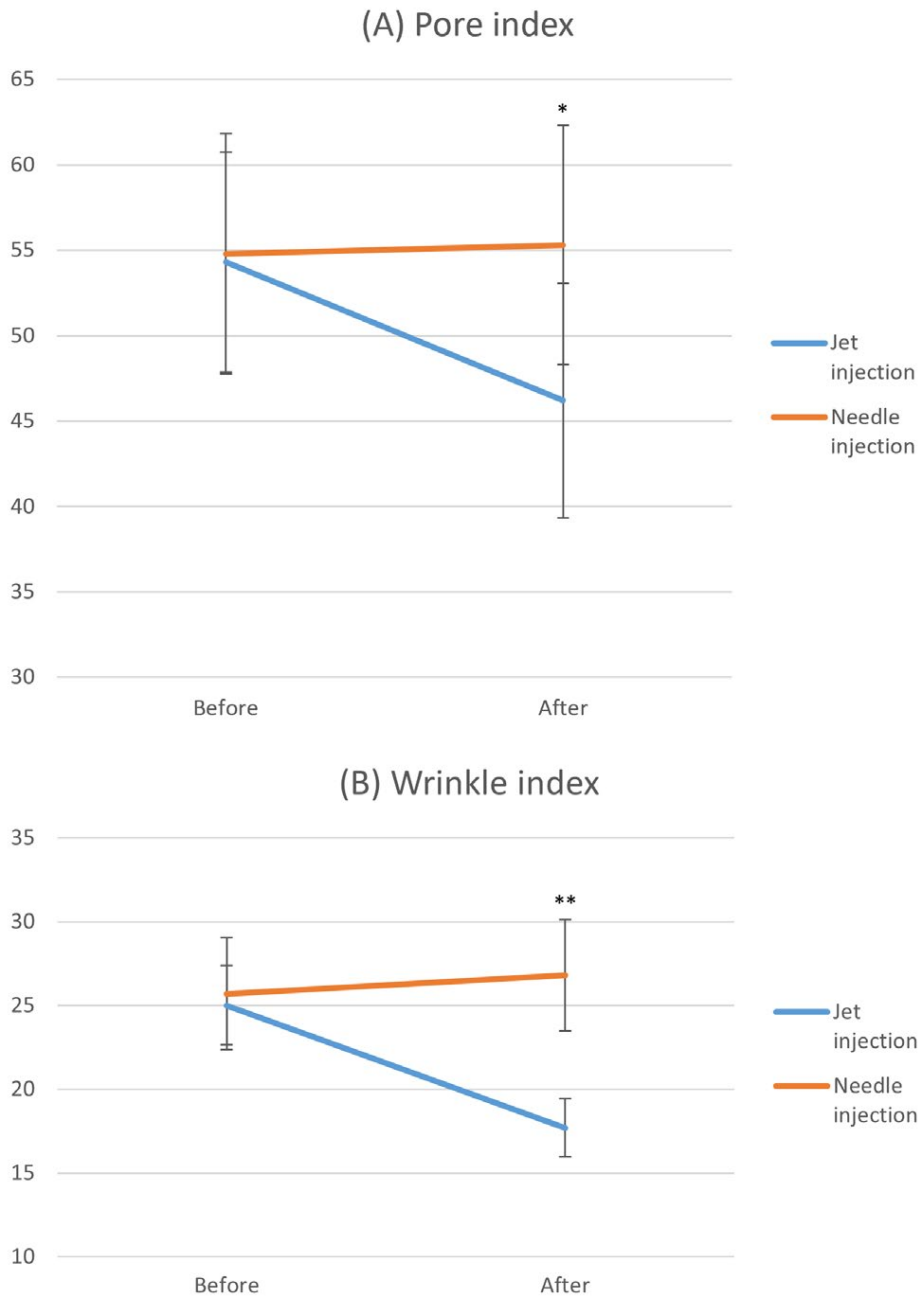
Improvement of (A) dilated pores and (B) wrinkles in the conventional injection and the needle‐free jet areas (*p < 0.05, **p < 0.01, and ***p < 0.001).

Skin parameters, including dilated pores, sebum levels, wrinkles, pigmentation, and skin brightness indices were evaluated using the three‐dimensional skin imaging tool Mark‐Vu. Notably, the pore and wrinkle indices showed significant improvement post‐treatment with CureJet, whereas manually injected areas, there was no pre‐and post‐treatment difference in these indices. In addition, while there was no difference between the two areas before treatment, the CureJet‐treated areas showed significant improvement compared to the manually injected areas for the pore index (*p* = 0.025) and wrinkle index (*p* = 0.006) as illustrated in Figure 2.

Table 2 details the Mark‐Vu Indices corresponding to these findings.

**TABLE 2:** Measurement of the Mark–Vu indices (mean ± SD) in the needle‐free jet areas and conventional injection area.

	Baseline	2 weeks after treatment	*p*‐value
A. Needle‐free jet areas			
Pore index	54.3 ± 6.44	46.2 ± 6.87	0.009**
Sebum index	10.1 ± 5.45	9.4 ± 4.59	0.39
Wrinkle index	25 ± 2.37	17.7 ± 1.73	< 0.001***
Pigmentation index	27.4 ± 9.69	28.1 ± 10.03	0.44
Skin brightness index	30.6 ± 8.13	28.8 ± 8.91	0.33
B. Conventional injection area			
Pore index	54.8 ± 7.04	55.3 ± 7.00	0.44
Sebum index	8.8 ± 4.49	8.5 ± 4.74	0.46
Wrinkle index	25.7 ± 3.35	26.8 ± 3.31	0.25
Pigmentation index	28.4 ± 9.34	27.6 ± 9.64	0.43
Skin brightness index	29.8 ± 10.98	28 ± 9.52	0.36

** *p* < 0.01.

*** *p* < 0.001

### 3.3 Safety Outcomes

Treatment‐related local adverse effects included mild, transient erythema in all patients, and focal bruising in the manual injection sites of two participants. These symptoms resolved spontaneously within a week post‐treatment. All patients tolerated the treatment well, with no serious side effects reported.

## 4 Discussion

**FIGURE 3:**
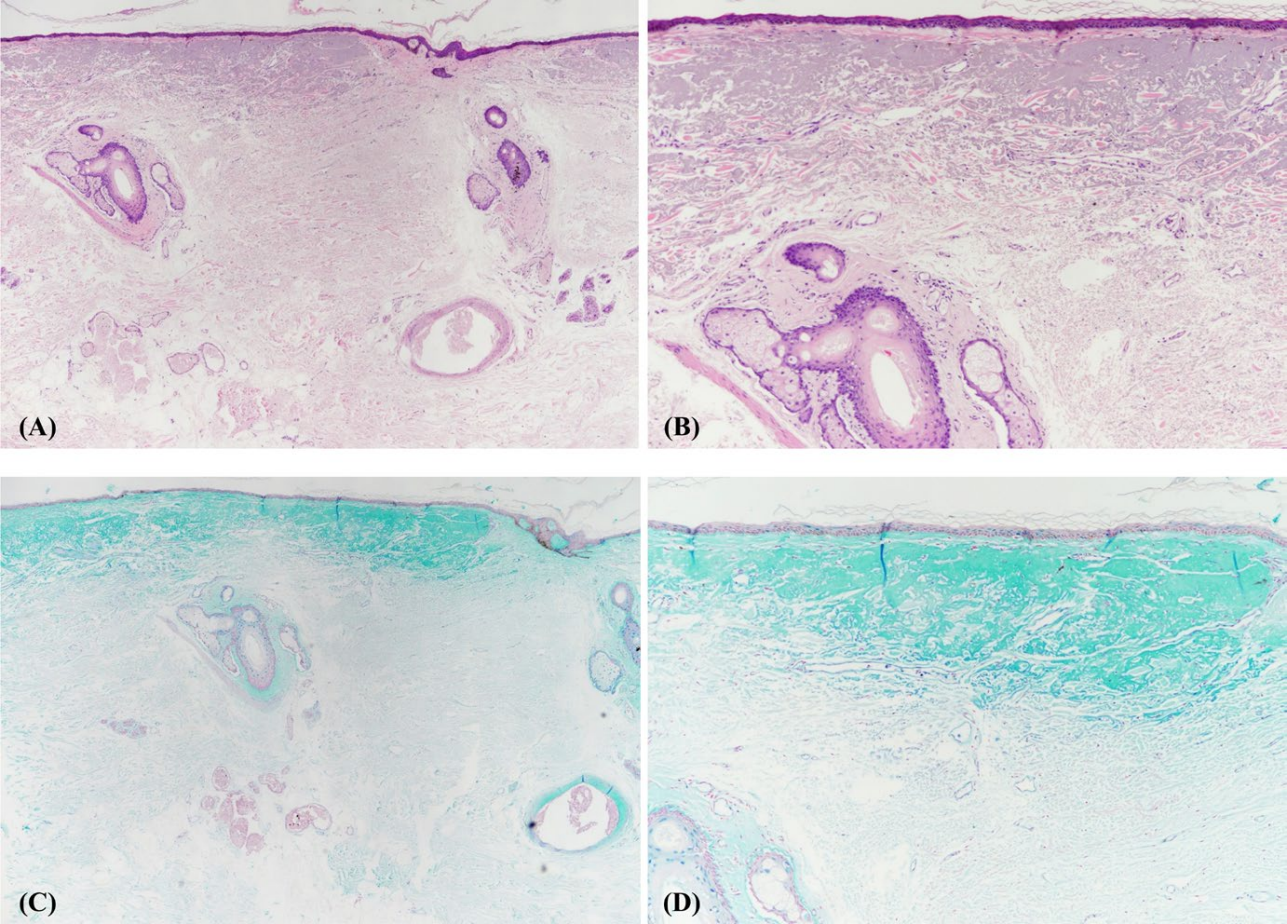
Histological samples showing poly D,L‐lactide and HA delivered through Curejet injection retained in the upper dermis. (A) H&E, ×40 (B) H&E, ×100 (C) Alcian blue, ×40 (D) Alcian blue, ×100.

Needle‐free injection systems are state‐of‐the‐art medical devices designed for delivering various medications, such as insulin and vaccines, into the skin without the need for traditional needle puncture [8]. The primary advantage of these systems is their ability to eliminate risks associated with skin puncture and disruption of the skin barrier [9]. This technology utilizes a needleless jet device, which administers a solution at high velocity by using pressurized air, facilitating its entry into the dermal layer. Although the procedure was performed on cadaver with a different type of filler, our previous experiments data (not shown) align with these findings, revealing that under our experimental settings, the drug was injected into and retained in the upper dermis (Figure 3) which suggest that a needleless jet device may be an effective way to target the upper dermis for filler injection. Since papillary fibroblasts exhibit greater proliferative and synthetic activities than reticular fibroblasts, but also show age‐related functional declines, targeted stimulation of the upper dermis using a needleless jet device may effectively enhance skin rejuvenation [10].

In our research, we observed that both the jet injection area and the conventional needle injection area exhibited improvement in GAIS, with the improvement being more significant in the jet injection area. In addition, the significant reduction in procedural pain in CureJet group resulted in overall patient satisfaction. This can help improving patient compliance to treatment and lowering psychological barrier toward injection. Also, remarkable improvement was observed in jet injection group, showing reduction of pore and wrinkles observed via magnification of MarkVu. This may be due to delicate positioning of injected materials by jet. Upon reaching the dermis, the liquid disperses evenly into the skin in a nearly spherical pattern, owing to the controlled deceleration of the jet at the exit point [11]. Over time, the orifice gradually narrows, simultaneously promoting dermal remodeling. This effect is achieved by stimulating fibroblasts through micro‐trauma, thus initiating neocollagenesis [12].

The limited sample size may have hindered the demonstration of a significant effect in the conventional needle injection area, while significant improvements can be observed in the jet injection area. This enhancement in the jet injection area is likely due to a synergistic effect resulting from the neocollagenesis induced by the jet injection.

Furthermore, there are also advantages over injection using needle such as the absence of side effects, including needle marks, bruising or bleeding, a lower risk of infection, and, most importantly, reduced pain during the procedure [13, 14]. Since the majority of pain occurs during the initial needle insertion, using a small opening in conjunction with rapid PN delivery through a pressurized device leads to a diminished sensation of pain during administration [14]. These benefits enhance patient compliance and make the treatment more comfortable to receive, as demonstrated by high levels of patient satisfaction scores.

The results of this study should be interpreted with an acknowledgment of some limitations. First, the small sample size limits the generalizability of the results. We could not determine potential differences according to skin types. Additionally, the experiment provided only short‐term follow‐up data, spanning a duration of 2 weeks, which may not be sufficient for a comprehensive assessment of the long‐term impacts. To validate the effectiveness and establish the most appropriate parameters, further controlled clinical studies involving a larger and more diverse patient population are imperative; acknowledging the limitation of the small sample size (*N* = 10), our future work will involve a more extensive clinical study with a significantly larger sample size. This will allow us to validate the preliminary results obtained from this pilot study and to perform more robust statistical analyses.

In conclusion, the needle‐free injection of PN for aging skin has demonstrated effectiveness in enhancing pore and wrinkle improvement while minimizing administration‐related discomfort. This approach may serve as a valuable alternative for patients with needle phobia, potentially working synergistically to promote neocollagenesis through the jet injection process.

## Conflicts of Interest

The authors declare no conflicts of interest.

## Data Availability

The data that support the findings of this study are available on request from the corresponding author. The data are not publicly available due to privacy or ethical restrictions.
